# Unidirectional neuronal cell growth and differentiation on aligned polyhydroxyalkanoate blend microfibres with varying diameters

**DOI:** 10.1002/term.2911

**Published:** 2019-06-11

**Authors:** Lorena R. Lizarraga‐Valderrama, Caroline S. Taylor, Frederik Claeyssens, John W. Haycock, Jonathan C. Knowles, Ipsita Roy

**Affiliations:** ^1^ Applied Biotechnology Research Group, School of Life Sciences, College of Liberal Arts and Sciences University of Westminster London UK; ^2^ Department of Materials Science and Engineering University of Sheffield Sheffield UK; ^3^ Division of Biomaterials and Tissue Engineering UCL Eastman Dental Institute London UK; ^4^ Department of Nanobiomedical Science and BK21 Plus NBM, Global Research Center for Regenerative Medicine Dankook University Cheonan South Korea; ^5^ The Discoveries Centre for Regenerative and Precision Medicine UCL Campus London UK; ^6^ UCL Eastman‐Korea Dental Medicine Innovation Centre Dankook University Cheonan South Korea

**Keywords:** electrospun fibres, nerve regeneration, peripheral nerves, polyhydroxyalkanoates, topographical guidance

## Abstract

Polyhydroxyalkanoates (PHAs) are a family of prokaryotic‐derived biodegradable and biocompatible natural polymers known to exhibit neuroregenerative properties. In this work, poly(3‐hydroxybutyrate), P(3HB), and poly(3‐hydroxyoctanoate), P(3HO), have been combined to form blend fibres for directional guidance of neuronal cell growth and differentiation. A 25:75 P(3HO)/P(3HB) blend (PHA blend) was used for the manufacturing of electrospun fibres as resorbable scaffolds to be used as internal guidance lumen structures in nerve conduits. The biocompatibility of these fibres was studied using neuronal and Schwann cells. Highly aligned and uniform fibres with varying diameters were fabricated by controlling electrospinning parameters. The resulting fibre diameters were 2.4 ± 0.3, 3.7 ± 0.3, and 13.5 ± 2.3 μm for small, medium, and large diameter fibres, respectively. The cell response to these electrospun fibres was investigated with respect to growth and differentiation. Cell migration observed on the electrospun fibres showed topographical guidance in accordance with the direction of the fibres. The correlation between fibre diameter and neuronal growth under two conditions, individually and in coculture with Schwann cells, was evaluated. Results obtained from both assays revealed that all PHA blend fibre groups were able to support growth and guide aligned distribution of neuronal cells, and there was a direct correlation between the fibre diameter and neuronal growth and differentiation. This work has led to the development of a family of unique biodegradable and highly biocompatible 3D substrates capable of guiding and facilitating the growth, proliferation, and differentiation of neuronal cells as internal structures within nerve conduits.

## 1 INTRODUCTION

Engineered scaffolds are designed to closely mimic the topography, spatial distribution, and chemical cues corresponding to the native extracellular matrix (ECM) of the intended tissue in order to support cell growth and differentiation. In tissue engineering, both three‐dimensional (3D) and two‐dimensional (2D) cell cultures are used. Porous scaffolds facilitate mass transfer and exchange of nutrients, metabolites, and gases. Additionally, their high surface area enhances cell adhesion and their interconnected porosity enables 3D cell ingrowth, which can be spatially controlled. Although the use of scaffolds with cocultures in 3D has been widely applied to regenerate a broad variety of tissues, these techniques have been scarcely used for nerve tissue regeneration. Three‐dimensional culture techniques would not only allow a better understanding of neuron–glial cell communication but could also contribute towards the development of scaffolds for peripheral nerve regeneration (Daud, Pawar, Claeyssens, Ryan, & Haycock, 2012).

The use of nerve guidance conduits (NGCs) to reconnect peripheral nerve gaps has been extensively investigated in the last 20 years. Notable efforts have been made to overcome the limitations of using the standard treatment, autografting, including donor site morbidity, scar tissue formation, scarcity of donor nerves, inadequate return of function, and aberrant regeneration. Although some NGCs made from natural and synthetic materials have been clinically approved, the regeneration obtained with them is only comparable with that using autologous grafts when the gaps are short (less than 5 mm). Commercial NGCs are all hollow tubes and can induce scar tissue and release compounds detrimental for the nerve regeneration process. Several research groups have investigated the introduction of structures within the lumen to improve neuronal regeneration such as luminal filaments, fibres, and multichannel structures (de Ruiter, Malessy, Yaszemski, Windebank, & Spinner, 2009; Jiang, Lim, Mao, & Chew, 2010).

Schwann cells are the glial cells of the peripheral nervous system. They insulate axons through wrapped layers of the myelin membrane, permitting and accelerating impulse conduction, compared with unmyelinated axons. It is well known that the two‐way communication between neurons and glial cells is crucial for normal functioning of the nervous system. Axonal conduction, synaptic transmission, and information processing are controlled by neuron–glial interaction. Neurons and glia communicate through cell adhesion molecules, neurotransmitters, ion fluxes, and specialized signalling molecules, whereas glial–glial cell communication relies on intracellular waves of calcium and intracellular diffusion of chemical messengers (Fields & Stevens‐Graham, 2002).

As the design of scaffolds should reproduce the tissue of interest, the native environment of neurons must be taken into consideration (Haycock, 2011). Although tissue‐engineered scaffolds may not exactly reproduce the target tissue, they have shown to provide a “nucleation structure” that trigger cellular self‐organization (Sun, Jackson, Haycock, & MacNeil, 2006). In tissue engineering, the main approach is to generate this “nucleating environment” in which 3D structures contain enough information for permitting cellular adhesion, proliferation, and differentiation, so the scaffold can become a mature and functioning construct. In the body, axons are surrounded by uniaxial aligned lipoprotein sheaths composed of myelin (Haycock, 2011). For this reason, research in scaffolds for nerve tissue engineering consists predominantly of 3D structures based on aligned fibres. Electrospinning is a versatile manufacturing method used to produce random or aligned fibres with either nanoscale or microscale diameters using a vast diversity of materials. Therefore, electrospinning is an ideal technique to reproduce aligned fibres to mimic the extracellular matrix environment of neuronal cells and serve as a nucleating environment. Additionally, this technique is also used to produce fibrous structures with random distribution, characteristic of the native ECM fibres found in majority of tissues such as the breast, liver, bladder, and lung (Cai, Yan, Liu, Yuan, & Xiao, 2013).

It has been shown that hollow NGCs are able to bridge nerve stumps resulting from severed nerves when the gaps are less than 10 mm by facilitating the formation of a fibrin cable. This fibrin cable supports the migration of Schwann cells permitting the recreation of longitudinally oriented bands of Büngner, which are aligned columns of Schwann cells and laminin. In this way, bands of Büngner serve not only as a source of neurotrophic factors but also as a guiding substrate that promotes axonal regrowth (Kim, Haftel, Kumar, & Bellamkonda, 2008).

In vitro cells have been shown to respond differently to diverse topographic scales, for example, nanoscale, microscale, and macroscales. Changes in the topography of a substrate can alter the biological behaviour due to different sensitivity scales of cells as a consequence of the variability in cell sizes, cell matrix, and filopodia (Sun et al., 2006). Several studies have demonstrated that aligned electrospun fibres can provide contact guidance to cultured cells, particularly leading to the elongation and alignment of cells along the axes of fibres. The resulting elongation along the fibres emulates the structure of bands of Büngner (Chew, Mi, Hoke, & Leong, 2007). Furthermore, it has been shown that aligned fibres are able to induce the orientation of focal adhesion contacts and the cell actin cytoskeleton through contact guidance (Badami, Kreke, Thompson, Riffle, & Goldstein, 2006).

A wide diversity of biodegradable materials has been used for the manufacturing of scaffolds for nerve tissue engineering applications. Fibres of both synthetic polymers (aliphatic polyesters, polylactic acids, and polycaprolactones [PCLs]) and natural polymers (gelatin and silk) have been already used as lumen modifications of NGCs. The use of biodegradable polymers for the manufacture of implants avoids a second surgery after implantation. After the implant is sutured at the nerve stumps, the scaffold is populated and remodelled by neuronal cells and eventually replaced by native tissue; hence, the original function can be restored. Although polyhydroxyalkanoates (PHAs), in particular poly(3‐hydroxybutyrate) (P(3HB)), have also been investigated for peripheral nerve regeneration applications (Hart, Wiberg, & Terenghi, 2003; Hazari, Wiberg, Johansson‐Rudén, Green, & Terenghi, 1999; Mohanna, Terenghi, & Wiberg, 2005; Mosahebi, Wiberg, & Terenghi, 2003; Mosahebi, Fuller, Wiberg, & Terenghi, 2002; Mosahebi, Woodward, Wiberg, Martin, & Terenghi, 2001; Ren et al., 2013), studies have focused on the fabrication of hollow NGCs without lumen modifications.

The aim of this work was to manufacture 25:75 poly(3‐hydroxyoctanoate) (P(3HO))/P(3HB) blend (PHA blend) electrospun fibres as resorbable scaffolds for their use in the manufacture of inner guidance fibres to be inserted in the lumen of NGCs. An in‐depth study on the effect of the PHA blend fibre diameter on cell growth and differentiation of NG108‐15 neuronal and RN22 Schwann cells was carried out. The choice of this particular PHA blend was driven by our work that we have published earlier (Lizarraga‐Valderrama et al., 2015), in which this blend was shown to be the most biocompatible with respect to neuronal cells when compared with the widely commercialized PCL and other P(3HO)/P(3HB) blend compositions. The biodegradation product resulting from the breakdown of P(3HB), 3‐hydroxybutyric acid, is a known natural metabolite found in the human body, hence is expected to have minimal toxicity and immunogenic response (Newman & Verdin, 2014). Also, the hydrolytic degradation of P(3HO) leads to the formation of 3‐hydroxyoctanoyl‐CoA, which is a natural metabolite found in the fatty acid beta oxidation pathway. Here, the enzyme 3‐hydroxyacyl‐CoA dehydrogenase can convert it to the corresponding enoyl‐CoA derivative (Houten & Wanders, 2010).

## 2 MATERIALS AND METHODS

### 2.1 Production and extraction of P(3HO) and P(3HB)

Production, extraction, and purification of P(3HO) and P(3HB) were performed as described previously (Rai, Keshavarz, Roether, Boccaccini, & Roy, 2011). The determination of lipopolysaccharides was carried out as described by Rai et al. (2011).

### 2.2 Film preparation

Films of PHA blends and PCL were prepared using the solvent‐casting method reported previously (Lizarraga‐Valderrama et al., 2015).

### 2.3 Scanning electron microscopy of films and scaffolds

Surface topography of the films and electrospun fibres was analysed using scanning electron microscopy (SEM) as described previously (Lizarraga‐Valderrama et al., 2015).

### 2.4 Manufacturing of aligned P(3HO)/P(3HB) blend fibres by electrospinning

Electrospinning was performed using a high voltage power supply (Genvolt, UK) with a syringe pump (WPI, USA) and a rotating cylindrical collector (IKA, UK). A 1‐ml plastic syringe (Intertronics, UK) with a blunt needle (20 G) connected to the power supply was used to fabricate the fibres. All the fibres were collected on a sheet of aluminium foil, which was used to wrap the electrically grounded collector. Aligned fibres of PHA blend were produced using varying polymer concentrations (15%, 25%, and 30% and 35% *w*/v) dissolved in chloroform under different voltage conditions (12 and 18 kV) and collector speed (1,500 and 2,000 rpm). All PHA solutions were electrospun at a distance of 10 cm from the collector for 1 min with a syringe pump flow rate of 1 ml/hr. The electrospun sheet dimensions after electrospinning were 5 cm × 20 cm, from which squares of 1.5 cm × 1.5 cm were removed for SEM analysis and cell culture experiments. Table 1 summarizes the processing conditions used to obtain PHA blend fibres with different diameters using varying polymer concentrations (15, 25, 30, and 35 wt%). Three representative diameters were chosen for cell culture studies on the basis of the uniformity in fibre size distribution: large (13.5 ± 2.3 μm), medium (3.7 ± 0.3 μm), and small (2.4 ± 0.3 μm).

**Table 1:** Summary of electrospinning conditions used to manufacture aligned P(3HO)/P(3HB) blend fibres of varying diameters using different polymer concentrations

Voltage (kV)	Collector speed (RPM)	Fibre diameter			
		Polymer concentration			
		15 wt%	25 wt%	30 wt%	35 wt%
12	2,000	2.4 ± 0.3	3.5 ± 0.3	4.3 ± 0.2	12.1 ± 3.6
	1,500	3.5 ± 0.4	4.1 ± 0.3	4.4 ± 0.3	13.5 ± 2.3
18	2,000	3.1 ± 0.3	3.4 ± 0.3	4.4 ± 0.8	14.0 ± 3.5
	1,500	3.4 ± 0.2	3.7 ± 0.3	5.1 ± 0.9	15.9 ± 8.0

### 2.5 Characterization of aligned P(3HO)/P(3HB) blend fibres by SEM

Three separated batches of samples belonging to the three fibre groups with different diameters were characterized using a Philips FEI XL30 Field Emission Gun Scanning Electron Microscope (Philips, Netherlands). Three parameters were analysed: (a) fibre diameter, (b) fibre density, and (c) fibre alignment. An average of 27 fibres per fibre group were studied for the determination of fibre diameter and fibre alignment. The same images used to determine parameters (a) and (c) were analysed to measure the density, which was defined as the number of fibres per unit area of the image. Fibre alignment was determined by measuring the angular variance between the fibres. Therefore, a reference line was drawn parallel to a central fibre, from which the angular difference was measured with respect to each fibre across the sample. The resulting data were collected in four groups classified by their angular difference (±0°, 1°, 2°, and 3°).

### 2.6 NG108‐15 neuronal cell culture

NG108‐15 neuronal cells were prepared as described previously (Daud et al., 2012).

### 2.7 Live/dead measurement of NG108‐15 neuronal cells

Live and dead cell measurements were carried out as previously reported (Daud et al., 2012).

### 2.8 Immunolabelling of NG108‐15 neuronal cells and RN22 Schwann cells

To assess the differentiation of neuronal cells, samples were immunolabelled using β‐III‐tubulin as the primary antibody and with Alexa Fluor^®^ 488 goat antimouse IgG as the secondary antibody (Sigma‐Aldrich, Gillingham, UK). The samples containing cultures of NG108‐15 neuronal cells were washed with phosphate buffered saline (PBS; ×3; Sigma‐Aldrich, Gillingham, UK) and fixed with 4% (*v*/v) paraformaldehyde for 20 min. Then they were permeabilized with 0.1% (*v*/v) Triton X‐100 (Sigma‐Aldrich, Gillingham, UK) for 20 min, before being washed with PBS (×3). Unreactive binding sites were blocked with 3% (*w*/v) bovine serum albumin (BSA; Sigma‐Aldrich, Gillingham, UK) with the cells being incubated overnight with mouse anti‐β‐III‐tubulin antibody (1:1000; Promega, Madison, USA) diluted in 1% BSA at 4°C. Cells were then washed three times with PBS before being incubated either with Alexa Fluor^®^ 488 goat antimouse IgG antibodies (1:200 in 1% BSA; Sigma‐Aldrich, Gillingham, UK) or Texas Red‐conjugated antimouse IgG antibody (1:100 dilution in 1% BSA) for 90 min (Sigma‐Aldrich, Gillingham, UK). After washing the cells once with PBS, 4′,6‐diamidino‐2‐phenylindole dihydrochloride (DAPI; 1:500 dilution in PBS; Sigma‐Aldrich, Gillingham, UK) was added to label nuclei along with phallodin‐FITC (1:1000 dilution in PBS; Sigma‐Aldrich, Gillingham, UK) to label RN22 Schwann cells when required. Cells were then incubated for 30 min at room temperature before being washed again with PBS (×3). Cells were then imaged using an upright Zeiss LSM 510 confocal microscope (Zeiss, Oberkochen, Germany) in the Kroto Research Institute imaging facility at University of Sheffield. Nuclei were visualized by two‐photon excitation using a Ti:sapphire laser (716 nm) for DAPI (*λ*_ex_ = 358 nm/*λ*_em_ = 461 nm; Sigma‐Aldrich, Gillingham, UK). For imaging the neuronal cell body and neurites, a helium–neon laser (543 nm) was used to detect Texas Red‐conjugated antimouse IgG antibody (1:100 dilution in 1% BSA; *λ*_ex_ = 589 nm/*λ*_em_ = 615 nm; Sigma‐Aldrich, Gillingham, UK) and an argon ion laser (488 nm) to detect Alexa Fluor^®^ 488 goat antimouse IgG (*λ*_ex_ = 495 nm/*λ*_em_ = 519 nm; Sigma‐Aldrich, Gillingham, UK). For imaging RN22 Schwann cell cytoskeleton, argon ion laser (488 nm) was used to detect phallodin‐FITC (*λ*_ex_ = 495 nm/*λ*_em_ = 521 nm; Sigma‐Aldrich, Gillingham, UK). Experimentally differentiated neuronal cells were then counted using ImageJ and identified as neuronal cells expressing neurites.

### 2.9 Statistical analysis

Statistical analysis was conducted using GraphPad Prism 6 software. A Shapiro–Wilk and Bartlett's test was previously performed to verify the normality and homogeneity of the data. To analyse the difference between data, a one‐way analysis of variance test (*p* < .05) was conducted followed by Tukey's posttest (*p* < .05). Data were reported as mean ± SEM.

## 3 RESULTS

### 3.1 Physical characterization of aligned PHA blend microfibres by SEM

**Figure 1:**
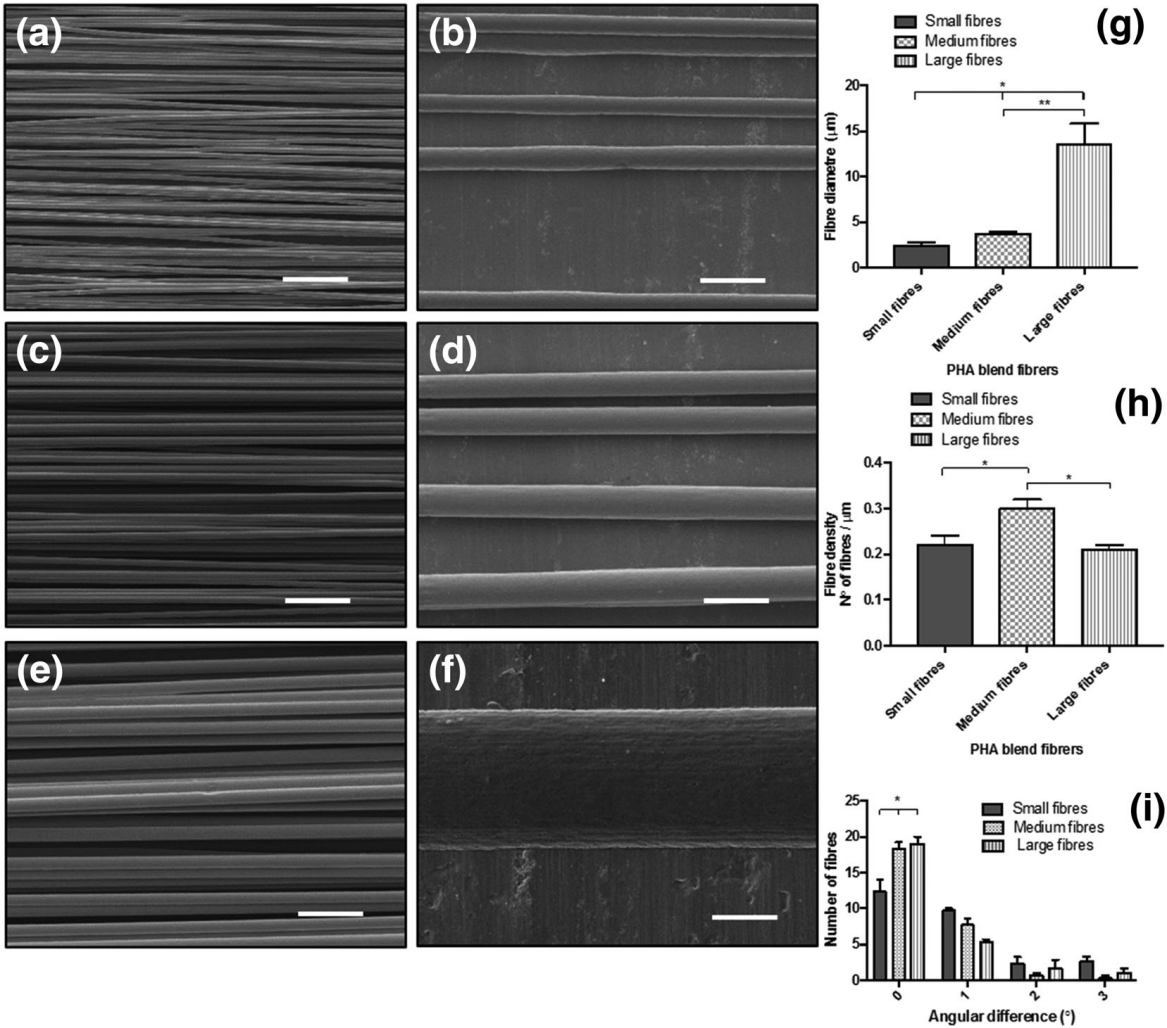
Characterization of electrospun PHA blend fibres by scanning electron microscopy. Diameter, density, and alignment of fibres were determined by image analysis. (a,b), (c,d), and (e,f) show the micrographs of the electrospun fibre mats corresponding to the small, medium, and large PHA blend fibre diameters, respectively. (g) Graph characterizing the fibre diameter for the different electrospinning conditions. The average diameters for the small, medium, and large fibres were 2.42 ± 0.34, 3.68 ± 0.26, and 13.50 ± 2.33 μm, respectively. A total of approximately 27 fibres were analysed for each fibre diameter group to determine the mean of the diameters (mean ± *SD*, *n* = 3 samples independently fabricated, ^*^ *p* < .05 compared with small fibres, ^**^ *p* < .05 in relation to medium fibres). (h) Mean fibre density measurement for each fibre group. The number of fibres contained in an area of 152 μm^2^ was measured (mean ± SEM, *n* = 3 samples independently fabricated). (i) Fibre alignment estimation. Angular difference between fibres and an assigned central reference fibre was measured. An average of 27 fibres was analysed for each fibre size group to determine the mean of each angular difference range (mean ± SEM, *n* = 3 samples independently fabricated, *p < .05 in comparison with medium fibres and large fibres). Scale bar = 50 μm

Three scaffolds consisting of aligned PHA blend microfibres with varying diameters were manufactured by electrospinning using three different processing conditions summarized in Table 1. The fibre architecture was studied using a field emission scanning electron microscope to analyse three physical features using the NIH ImageJ software: (a) fibre diameter, (b) fibre density, and (c) fibre alignment. SEM micrographs of small, medium, and large fibres are shown in Figure 1a,b; Figure 1c,d; and Figure 1e,f, respectively. After analysis of the scaffolds, the resulting average fibre diameters for small, medium, and large fibre groups were measured to be 2.4 ± 0.3, 3.7 ± 0.3, and 13.5 ± 2.3 μm, respectively (Figure 1g). Statistical analysis showed that the difference in fibre diameter between the small and medium fibre group compared with the large fibre group was significant (2.4 ± 0.3 vs. 13.5 ± 2.3 μm, ^*^ *p* < .05, and 3.7 ± 0.3 vs. 13.5 ± 2.3 μm, ^**^ *p* < .05).

Fibre density determination was also carried out on the basis of the SEM micrograph images. It can be seen in Figure 1h that the medium‐sized fibre group (0.30 ± 0.03 fibres per micrometre) exhibited the highest fibre density compared with the small (0.22 ± 0.04 fibres per micrometre, ^*^ *p* < .05) and the large fibre groups (0.21 ± 0.02 fibres per micrometre, ^**^ *p* < .05). However, no significant difference was found between the small and large fibre groups.

Fibre alignment estimation was assessed by measuring the angular difference between the fibres and an assigned central reference line. For each size fibre group, an average of 27 fibres was analysed to determine the mean of each angular difference group and is presented in a histogram (Figure 1i**)**.

A major proportion of fibres for the three size groups presented an angular difference of 0°. Medium and large fibres showed the highest number of fibres with an angular difference of 0° when compared with small fibres (^*^ *p* < .05 in comparison with medium fibres and large fibres). The proportion of fibres with increasing angle of variance dropped markedly, showing that the majority of the fibres were arranged in a straight line and parallel to each other (Figure 1a,c,e,h).

### 3.2 Live/dead measurement of NG108‐15 neuronal cells on PHA blend electrospun fibres

**Figure 2:**
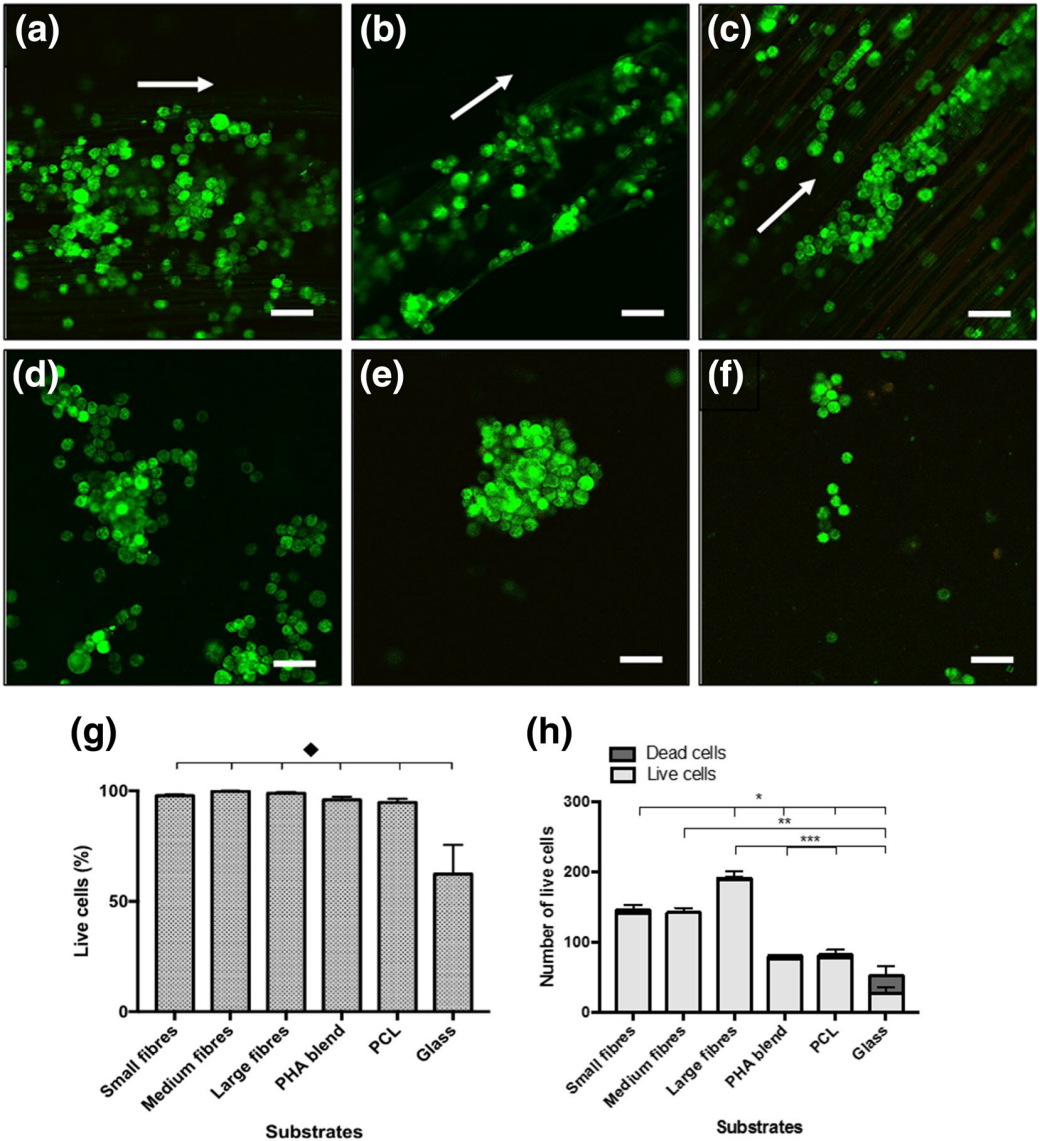
Confocal micrographs of NG108‐15 neuronal cells labelled with propidium iodide (red; dead cells) and Syto‐9 (green; live cells) after 4 days in culture on aligned PHA blend fibres and live/dead cell analysis. (a) Small fibres (2.42 ± 0.34 μm). (b) Medium fibres (3.68 ± 0.26 μm). (c) Large fibres (13.50 ± 2.33 μm). (d) PHA blend film. (e) PCL film. (f) Glass. Scale bar = 50 μm. (g) Live/dead analysis of neuronal cells on fibres with varying diameters and controls. Percentage of live neuronal cells on the fibres, PHA blend film, and PCL film was higher in comparison with glass (control; mean ± SEM, *n* = 9 independent experiments, ^♦^ *p* < .05). (h) Histograms showing numbers of neuronal cells on fibres with varying diameters and controls [Colour figure can be viewed at http://wileyonlinelibrary.com]

Live/dead cell assays were performed in order to compare the attachment and survival of NG108‐15 neuronal cells on the electrospun PHA blend sheets with varying fibre diameters. In Figure 2, representative confocal images of neuronal cells grown on different substrates are shown.

Figure 2a–c corresponds to cells grown on the small, medium, and large PHA blend fibres, respectively. It can be seen that neuronal cell growth had an aligned distribution on all three fibre groups (Figure 2a–c). On the other hand, random growth of cells was observed on the flat substrates of PHA blend (Figure 2d), PCL (Figure 2e), and glass (Figure 2f), in which several clusters of cells were found.

The percentage of live cells on small fibres (97.76 ± 0.58%), medium fibres (99.77 ± 0.22%), large fibres (98.87 ± 0.60%), flat PHA blend film (95.89 ± 1.43%), and flat PCL film (94.72 ± 1.68%) was higher when compared with glass (62.28 ± 13.16%, ^♦^ *p* < .05; Figure 2g). However, no significant differences in percentage of live cells were found between the fibre groups. The number of neuronal cells that grew on the small (142.20 ± 11.42; *p < .05), medium (142.67 ± 6.36, **p < .05), and large (188.58 ± 13.00, ^***^ *p* < .05) fibre groups was higher when compared with that obtained on the glass control (27.20 ± 8.40; Figure 2h).

As seen in Figure 2h, the number of neuronal cells that adhered to and grew on the small (142.20 ± 11.42, *p < .05) and large (188.58 ± 13.00, ***p < .05) fibre groups was higher when compared with that obtained on the PHA blend (76.89 ± 5.60) and PCL (78.00 ± 12.04) flat substrates (Figure 2h). No significant difference was found between the number of cells grown on medium fibres (140.30 ± 10.22) when compared with those grown on either small or large fibres. Large fibres supported the highest number of neuronal cells (188.58 ± 13.00) when compared with the controls (PHA blend, PCL flat substrates, and glass, ***p < .05) and small fibres (*p < .05). The number of cells grown on medium fibres was only significantly different to the number of cells grown on glass. No significant difference was found between the number of neuronal cells grown on the controls (PHA blend, PCL, and flat substrates and glass).

### 3.3 Neurite outgrowth assessment on NG108‐15 neuronal cell culture grown on PHA blend electrospun fibres

**Figure 3:**
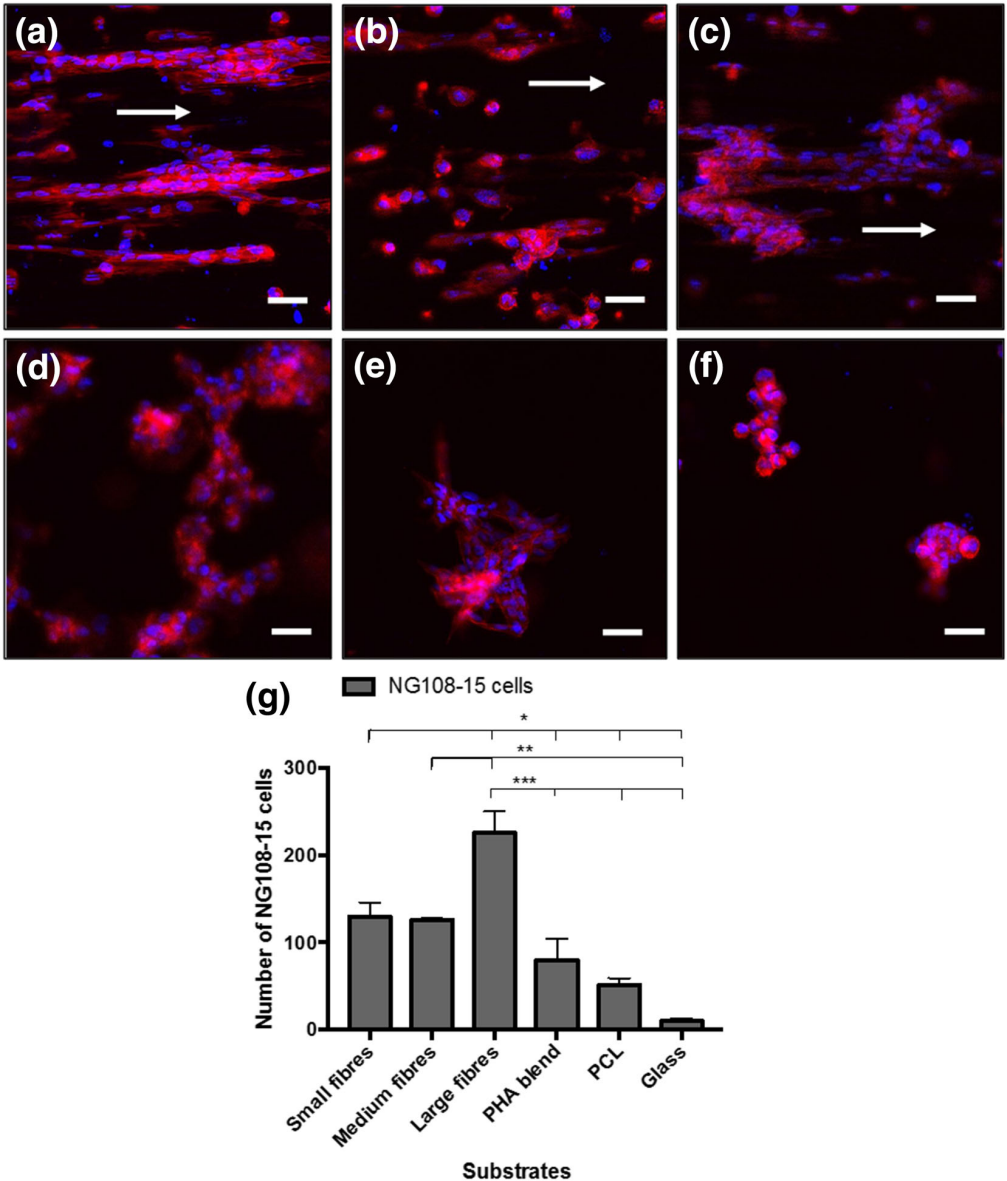
Confocal micrographs of NG108‐15 neuronal cells inmunolabelled for β‐III‐tubulin after 4 days in culture on aligned PHA blend fibres, PHA, PCL flat substrate, and glass. Nuclei are counterlabelled with DAPI. (a) Small fibres (2.42 ± 0.34 μm). (b) Medium fibres (3.68 ± 0.26 μm). (c) Large fibres (13.50 ± 2.33 μm). (d) PHA blend film (flat substrate). (e) PCL film (flat substrate). (f) Glass. Scale bar = 50 μm. (g) Number of neuronal cells with neurites on P(3HO)/P(3HB) blend fibres, PCL, and glass (control; mean ± SEM, *n* = 9 independent experiments, *p <* .05). The number of differentiated cells grown on the large fibre substrate was significantly higher when compared with the small fibre substrate and controls (^*^ *p* < .05). The number of neuronal cells on medium fibres was significantly different only when compared with that on large fibres and controls (^**^ *p* < .05). The number of differentiated neuronal cells that grew on large fibres was significantly different when compared with the rest of electrospun fibrous substrates (small and medium) and flat substrates (PHA blend, PCL, and glass; ^*^ *p* < .05, ^**^ *p* < .05, ^***^ *p* < .05) [Colour figure can be viewed at http://wileyonlinelibrary.com]

NG108‐15 neuronal cells were grown on the scaffolds and immunolabelled with the anti‐β III‐tubulin antibody to assess neurite outgrowth and differentiation. Figure 3a–f shows the confocal images of immunolabelled neuronal cells grown on all the substrates.

Differentiation was observed in all the neuronal cells that were positive for anti‐β‐III‐tubulin antibody in all the substrates including the controls: PHA blend (Figure 3d), PCL (Figure 3e), and flat substrates and glass (Figure 3f). Alignment of cell growth was clearly seen on the three fibre groups—small fibres (Figure 3a), medium fibres (Figure 3b), and large fibres (Figure 3c)—whereas a random distribution of cells was observed in the controls: PHA blend (Figure 3d), PCL (Figure 3e), and flat substrates and glass (Figure 3f).

Figure 3g shows the number of differentiated neuronal cells on each substrate. The large fibre group displayed the highest number of differentiated neuronal cells (225.67 ± 24.85) compared with the small (129.13 ± 16.58, *p < .05) and medium (125.33 ± 2.40, **p < .05) fibre groups. Statistical analysis showed no significant difference between the number of neuronal cells that grew on the small (129.13 ± 16.58) and medium (125.33 ± 2.40) fibre groups. The number of differentiated neuronal cells on the small (129.13 ± 16.58, *p < .05) and large (225.67 ± 24.85, ***p < .05) fibre groups was found significantly different to those measured on the flat substrates, the PHA blend (79.88 ± 24.49), PCL (51.25 ± 7.87), and glass (10.13 ± 2.96). However, no significant difference was found between the number of differentiated neuronal cells found on the medium fibre group with respect to PHA blend and PCL flat substrates (Figure 3). No significant difference was found between any of the flat substrates, PHA blend, PCL, and glass. In Figure S1, differentiated neuronal cells grown on the three fibre groups are shown with a higher magnification (40×). Neurite‐bearing cells can be observed forming parallel aligned groups of cells in the three fibre groups: small fibre (Figure S1A–J), medium fibre (Figure S1B–K), and large fibre (Figure S1C–L). On the other hand, clusters of cells were observed on the controls, PHA blend (Figure S2A,D), PCL (Figure S2B,E), and flat substrates and glass (Figure S2C,F)

### 3.4 Neurite outgrowth assessment on NG108‐15 neuronal cell/Schwann cell cocultures grown on PHA blend electrospun fibres

**Figure 4:**
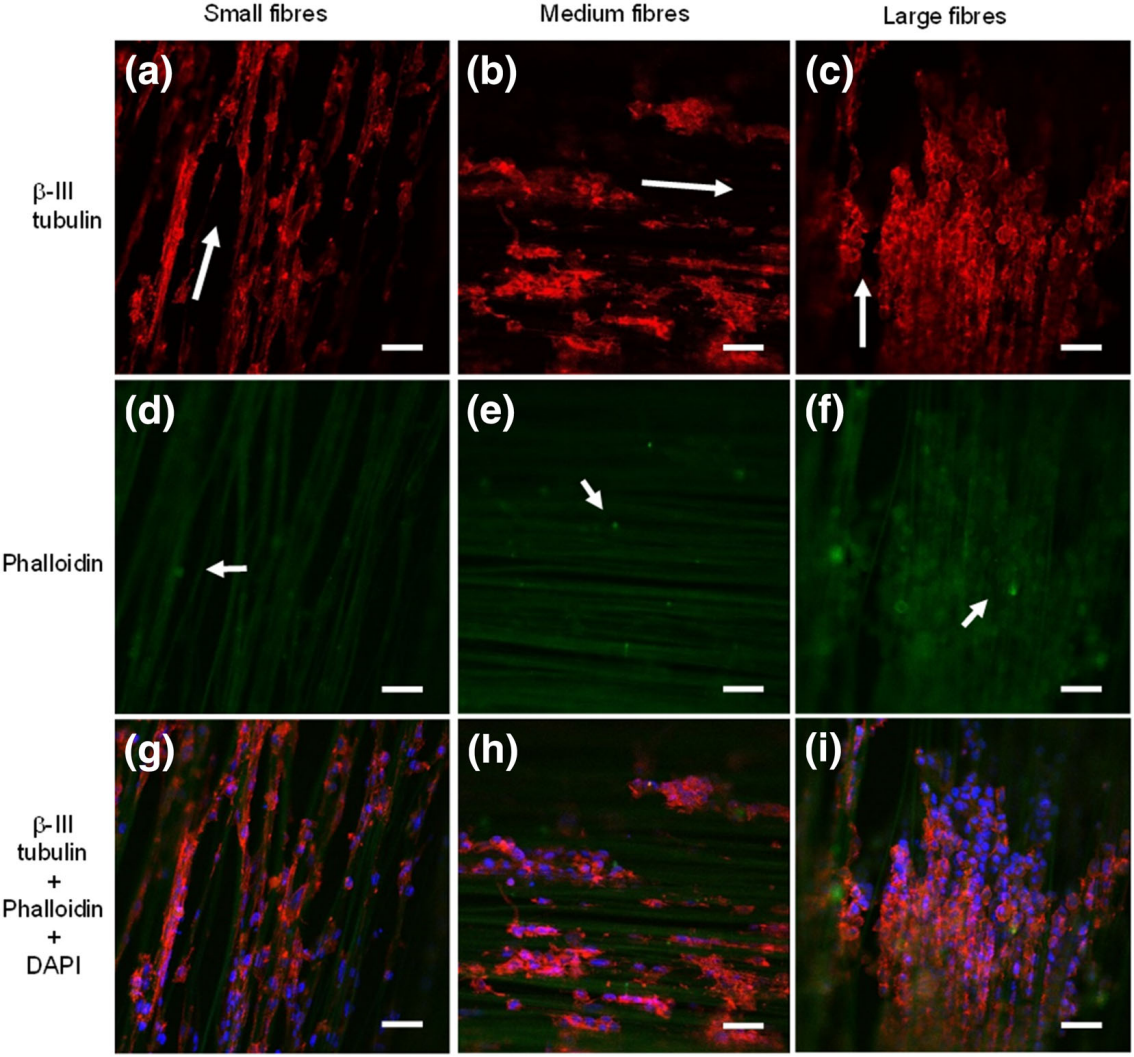
Confocal micrographs of NG108‐15 neuronal cells (red) grown with RN22 Schwann cells (green) inmunolabelled for β‐III tubulin and stained with phalloidin and DAPI after 4 days in culture on aligned PHA blend fibres. (a,d,g) Small fibres (2.42 ± 0.34 μm), (b,e,h) medium fibres (3.68 ± 0.26 μm), and (c,f,i) large fibres (13.50 ± 2.33 μm). Alignment of neuronal cells was observed in all three fibre groups. Scale bar = 50 μm [Colour figure can be viewed at http://wileyonlinelibrary.com]

**Figure 5:**
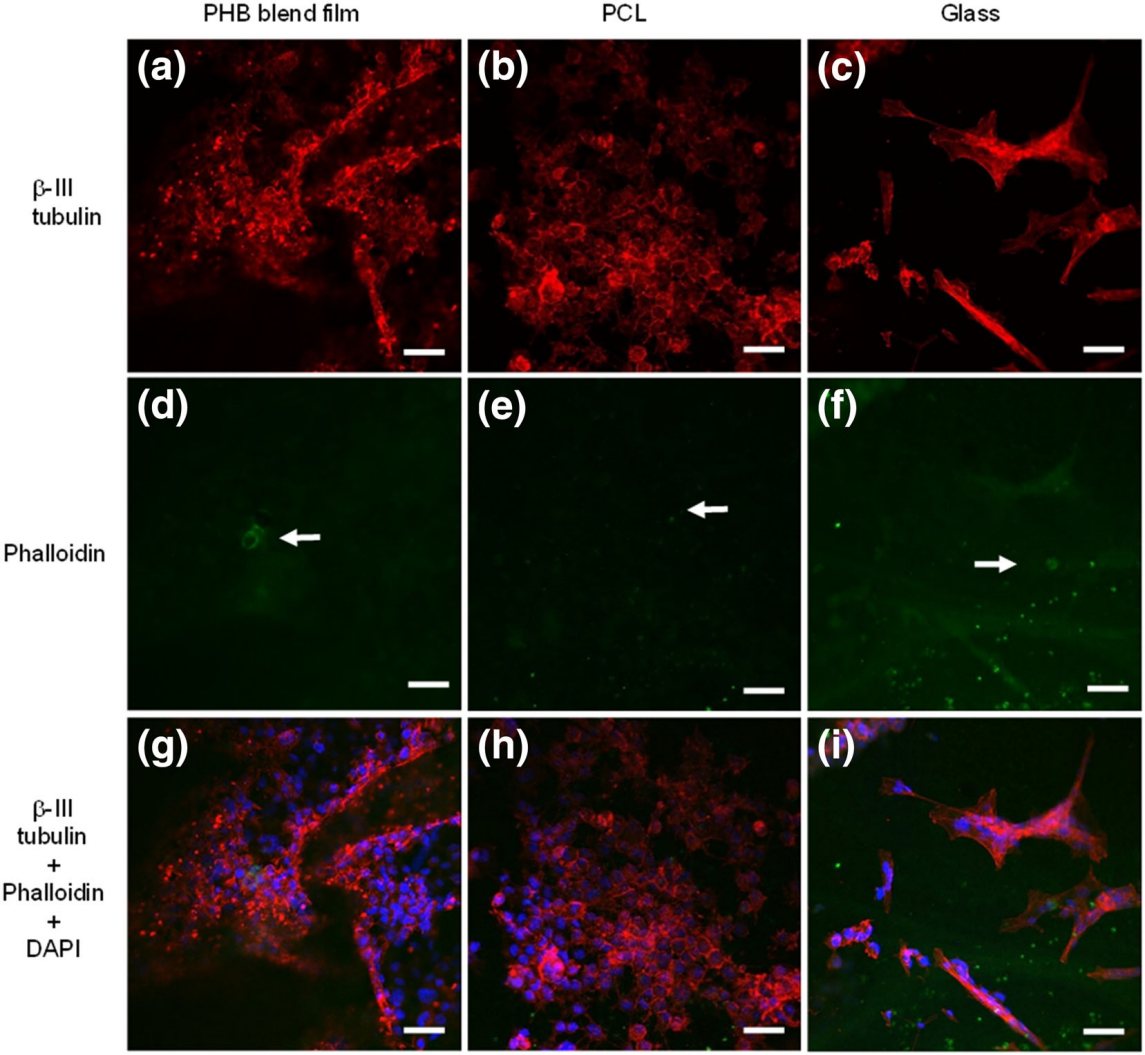
Confocal micrographs of NG108‐15 neuronal cells (red) grown with RN22 Schwann cells (green) inmunolabelled for β‐III tubulin and stained with phalloidin (F‐actin) and DAPI (nuclei), after 4 days in culture on flat substrates (controls) consisting of (a,d,g) PHA blend film, (b,i,h) PCL film, and (c,f,i) glass. Cell growth was randomly oriented on the flat substrates, the PHA blend film, PCL film flat substrates, and glass. Scale bar = 50 μm [Colour figure can be viewed at http://wileyonlinelibrary.com]

Confocal micrograph images of NG108‐15 neuronal cells (red) grown in coculture with RN22 Schwann cells (green) on small fibres (Figure 4a,d,g), medium fibres (Figure 4b,e,h), and large fibres (Figure 4c,f,i) are shown in Figure 4. Only a few Schwann cells were detected after 4 days in coculture and were observed to attach to all three fibre groups: small fibres (Figure 4d), medium fibres (Figure 4e), and large fibres (Figure 4f). Although only a few Schwann cells were detected, the growth of neuronal cells confirmed that these two cell lines were able to coexist on all the fibre groups. Alignment of neuronal cell growth was observed in all fibre groups: small fibres (Figure 4a), medium fibres (Figure 4b), and large fibres (Figure 4c). However, random growth was observed on the control substrates: PHA blend (Figure 5a), PCL (Figure 5b), and flat substrates and glass (Figure 5c). After analysis of confocal images using ImageJ, all neuronal cells were found to be differentiated on all the substrates: small fibres (Figure 4g), medium fibres (Figure 4h), large fibres (Figure 5i), PHA blend (Figure 5g), PCL (Figure 5h), and flat substrates and glass (Figure 5i).

**Figure 6:**
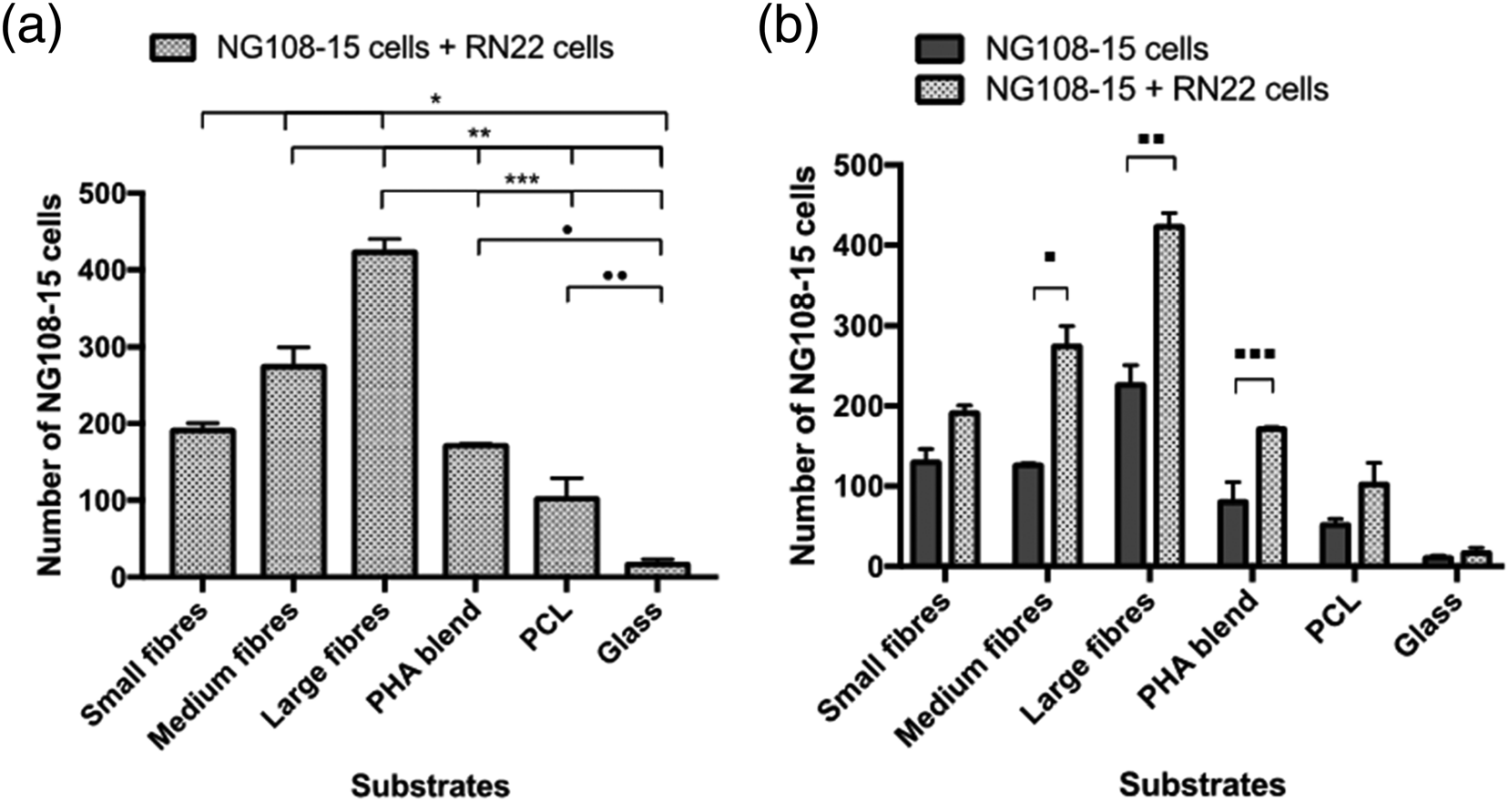
Number of neuronal cells grown with Schwann cells on all the substrates. Statistical analysis showed that the numbers of neuronal cells found on the three fibre groups was significantly different. Also, the number of neuronal cells grown on the fibre groups increased as the fibre diameter increased (Figure 6a). The number of neuronal cells found on large fibres (423.25 ± 16.90) was significantly higher than those found on small (191.00 ± 9.50, ^*^ *p <* .05) and medium fibres (274.43 ± 25.36, ^***^ *p <* .05; Figure 6a). (b) Number of neuronal cells grown on their own compared with number of neuronal cells grown in coculture with Schwann cells

Similarly, the number of NG108‐15 neuronal cells that adhered and grew on the medium fibres (274.43 ± 25.36) was higher than that measured on small fibres (191.00 ± 9.50, ^*^ *p <* .05; Figure 6a). The resulting number of neuronal cells found on the medium (^**^ *p <* .05) and large fibre groups (^***^ *p <* .05) was significantly higher than those measured on the control substrates: PHA blend, PCL flat substrates, and glass. Significant differences were also found in the number of neuronal cells grown on the P(3HO)/P(3HB) blend (171.00 ± 2.65, ^•^ *p <* .05) and PCL (102.00 ± 27.13 ^••^ *p <* .05) flat substrates with respect to glass (16.67 ± 7.04). Figure 6b shows the number of neuronal cells grown on all substrates in coculture with Schwann cells versus the number of neuronal cells grown on all the substrates without Schwann cells. Interestingly, statistical analysis showed that the number of neuronal cells grown on medium fibres was significantly higher when these cells were grown in coculture with Schwann cells (274.43 ± 25.36) compared with the number of NG108‐15 grown on their own (125.33 ± 2.40, ^■^ *p <* .05; Figure 6b). Similarly, the number of neuronal cells grown on large fibres (225.6 ± 24.85) increased when they were grown with Schwann cells (423.25 ± 16.90, ^■■^ *p <* .05; Figure 6b).

The number of neuronal cells measured on the P(3HO)/P(3HB) blend control (79.88 ± 24.49) was also significantly different when grown along with RN22 (102.00 ± 27.13, ^■■■^ *p <* .05; Figure 6b). No significant increment in the number of neuronal cells was found when NG108‐15 were cocultured with RN22 cells on small fibres (129.13 ± 16.58 vs. 191.00 ± 9.50), PCL (51.25 ± 7.87 vs. 102.00 27.13), and glass (10.13 ± 2.96 vs. 16.67 ± 7.04; Figure 6b).

## 4 DISCUSSION

This investigation shows that the development of a 3D scaffold consisting of aligned microfibres results in the successful alignment of neuronal cell growth either individually or in coculture with RN22 Schwann cells. Because PHA blends had previously been shown to display superior biocompatibility with neuronal NG108‐15 cells compared with the commercial PCL, other PHA blends, and neat PHAs, this blend was chosen for the fabrication of microfibres to be used for lumen modification of NGCs. Several fibre diameters were produced using PHA blends by electrospinning in order to assess the effect of fibre diameter on the response of neuronal cells.

Three‐dimensional cultures provide an effective approach to develop a functional construct that mimics the natural architecture of a multicellular/tissue environment. In their native environment, the majority of cells are surrounded by an extracellular matrix together with phenotypically dissimilar stromal cells in a 3D fashion. Three‐dimensional scaffolds should be able to provide physical cues that promote topographical stimuli to trigger cell adhesion, growth, and differentiation. Also, the scaffold should be designed to permit the exchange of vital nutrients and gases with the external environment. Development of 3D cultures is rapidly gaining relevance as they mimic more accurately the native environment of cells compared with 2D cultures.

Drug discovery research has driven a considerable amount of innovations in 3D culture systems over the past 5 years (Edmondson, Broglie, Adcock, & Yang, 2014). Also, tissue engineering, cancer, and stem cell research have adopted and adapted 3D culture methods to obtain more predictable in vivo data. It has been shown that cell responses to 2D and 3D environments are different. Focal adhesion, proliferation, population, differentiation, and gene and protein expression have been shown to be different for the same cell type when grown on a 3D scaffold compared with that on a 2D construct (Sun et al., 2006). In fact, it has been shown that cells in the 3D culture environment differ morphologically and physiologically from cells grown in a 2D culture environment (Edmondson et al., 2014). To date, 3D cultures have been used to study more than 380 cell lines (Ravi, Paramesh, Kaviya, Anuradha, & Paul Solomon, 2015). However, most of cell studies are still based on the use of 2D cultures using flat and rigid substrates, which considerably differ from the native environment of the cells. As a result, it has been shown that 2D cell culture assays can provide misleading and nonpredictive data for in vivo cell behaviour.

Three‐dimensional cultures influence signal transduction by affecting the spatial organization of the cell surface receptors involved in cell interaction. This will ultimately induce a specific gene expression profile, resulting in a particular cell behaviour (Edmondson et al., 2014). In spite of the fact that limitations of 2D cultures are well known, only few 3D models have been developed for nerve regeneration research. A further reduced number of studies are based on the use of coculture models using natural polymers to investigate peripheral nerve repair with the ultimate aim of manufacturing NGCs. Several scaffolds have been developed for peripheral nerve regeneration research including porous matrices, random fibres, and hydrogels. However, only few studies have described the use of aligned fibres using more than one cell type (Daud et al., 2012; Wang, Mullins, Cregg, McCarthy, & Gilbert, 2010). In the majority of the studies, aligned fibres are used and have focused on the use of a single cell type such as primary neurons extracted from dorsal root ganglia (Corey et al., 2007), PC12 cells (Mohanna et al., 2005), and primary human Schwann cells isolated from sciatic nerves of human foetuses (Behbehani et al., 2018; Chew et al., 2007).

Previous studies have shown that the diameter of fibres can affect cell growth and function not only in neuronal cells (Gnavi et al., 2015) but also in different cell types such as human dermal fibroblasts (Liu et al., 2009) and osteoblasts (Badami et al., 2006). In the case of peripheral nerve repair, it is still not clear whether nanofibres or microfibres support better nerve regeneration. Some researchers argue that nanofibres should be better scaffolds because their dimensions and architecture are closer to the native structure of the extracellular matrix of neurons (Jiang, Mi, Hoke, & Chew, 2014). Nevertheless, comparative studies are not conclusive and have shown that despite the resemblance of nanofibres to the ECM of neurons, they have not displayed a satisfactory outcome. Optimal results in nerve regeneration have been reported by using both nanofibres (Wang et al., 2010; Wang et al., 2011) and microfibres (Hurtado et al., 2011); however, studies of fibre size effect in nerve regeneration remain limited. In fact, Yao et al. (2009) fabricated PLLA fibres of varying diameters and found that neurite outgrowth and cell migration were inhibited when fibres of 200‐nm diameter were used. Conversely, Wen and Tresco found good alignment and outgrowth of neurites on microfibres with diameters ranging from 30 μm (comparable with cellular size) to 5 μm. However, Schwann cell migration and neurite outgrowth were inhibited when the diameter of the microfibres was greater than 30 μm (Wen & Tresco, 2006). In an in vitro study, small‐diameter fibres resulted in a decreased neurite length of 42% and 36% compared with the large (1,325 + 383 nm) and intermediate (759 + 179 nm) diameter fibres, after 5 days of culturing chick dorsal root ganglion (DRG; Wang et al., 2010). However, in an in vivo study, nanofibres exhibited better results compared with microfibres when used as lumen scaffolds in NGCs for repairing 15‐mm critical defect gaps. Nanofibre conduits (251 ± 32 nm) promoted a significantly higher number of myelinated axons and thicker myelin sheaths compared with microfibre conduits (981 ± 83 nm). Additionally, nanofibre conduits produced an increased number of regenerated DRG sensory neurons (1.93 ± 0.71 × 10^3^) compared with microfibre conduits (0.98 ± 0.30 × 10^3^; Jiang et al., 2014). Conversely, in a 3D in vitro model, microfibres with higher diameter (8 μm) supported a better outcome of neurites outgrowth than fibres with 5‐ and 1‐μm diameter. However, when neurons were grown in coculture along with Schwann cells or as DRG explants, the smallest fibres (1 mm) displayed superior performance in neurite outgrowth and Schwann cell migration (Daud et al., 2012). In the present study, highly aligned large fibres (13.50 ± 2.33 μm), which displayed the best performance of neurite outgrowth, resemble α‐fibres in diameter (12–22 μm; Woessner, 2006), replicating to some extent the microtopography that surrounds axon fibres inside the fascicles. Despite the extensive development of manufacturing techniques in the field of tissue engineering, the complex mixture of nanotopography and microtopography characterizing the neuronal cellular environment has not yet been replicated.

In this investigation, highly aligned and uniform PHA blend fibres with varying diameters were successfully fabricated by controlling electrospinning parameters summarized in Table 1. In a preliminary study, the effect of fibres on the growth of NG108‐15 neuronal cells was investigated by a live/dead test. Cell growth and migration observed on the electrospun fibres showed directional alignment in accordance with the direction of the fibres. The distribution of cells on all the electrospun fibre sizes had a more uniform arrangement when compared with that on the surface of the PHA blend film. This finding agreed with previous studies in which electrospun fibres have been shown to affect cell proliferation, differentiation, and migration (Daud et al., 2012). No cytotoxic effect was observed when neuronal cells were grown on any of the studied substrates. Thereafter, the correlation between PHA blend microfibre diameter and neuronal growth under two conditions, individually and in coculture with RN22 Schwann cells, was evaluated. This was investigated using two types of cell staining, live/dead cell test and anti‐β tubulin immunolabelling, to identify neuronal neurite formation. Results from both studies revealed that all PHA blend fibre groups were able to support growth well and to guide aligned distribution of neuronal cells when grown individually and in the presence of RN22 Schwann cells. All the substrates, including the controls, were found to support neurite outgrowth but with different levels of efficiency. Electrospun fibre substrates were shown to support better both cell growth and differentiation. Although the counting of the neurite bearing cells was not possible, higher abundance of neurite bearing cells was observed in the fibrous scaffolds (Figure S1). Although the electrospinning technique allows optimal fabrication of aligned fibres and there are significant benefits to having tissue engineering constructs at the length scales, its commercial use is limited due to poor replicability and constraints for scaling‐up. Furthermore, the need of high voltages and formation of random alignment during the process confine this technique to the academic niche. An innovative technique, pressurized gyration, established in 2013, could be used to produce more replicable aligned fibres and at much higher throughput, for applications in the field of nerve tissue engineering. Pressurized gyration benefits from an increased number of parameters that can be modified for manufacturing large quantities of homogenous fibers, resulting in a greater control over the final morphology (Heseltine, Ahmed, & Edirisinghe, 2018).

Results revealed a direct correlation between fibre diameter and neuronal growth and differentiation. Although neuronal cell viability was similar for all the substrates (approximately 99%) except on glass, large fibres supported the highest number of live neuronal cells grown individually compared with the rest of substrates. However, no significant difference was found between the number of live neuronal cells grown on small fibres and medium fibres. A similar outcome was found when neuronal cell differentiation was assessed in the single cell type culture. The greatest extent of neuronal cell differentiation was displayed on large fibres, whereas no significant difference was found between small and medium fibres (Figure 6a). Interestingly, when neuronal cells were grown in coculture with RN22 Schwann cells, the number of NG108‐15 cells increased as the fibre diameter increased (Figure 6b). These findings correlated with previous studies in which variation on the diameter of electrospun fibres made affected the neurite outgrowth (Wang et al., 2010). Ren et al. (2013) found an enhanced differentiation of human neural crest stem cells towards the Schwann cell lineage when an aligned electrospun fibre matrix was used as the substrate. In a similar study, Du et al. (2017) found rapid directional cell adhesion and migration of both Schwann cells and DRGs grown on aligned electrospun nanofibre hydrogel matrix. The aligned topography of the matrix accelerated axonal cell outgrowth when compared with a random fibre matrix (Du et al., 2017). Wang et al. (2011) grew human embryonic stem cells on Tussah silk fibroin scaffold using both random and aligned orientation with diameters of 400 and 800 nm, to study the effect of fibre alignment and diameter on cell viability and neuronal differentiation. They found that the aligned Tussah silk fibroin scaffold with 400‐nm fibres displayed the best neuronal differentiation (Wang et al., 2010). In a similar study, Panahi‐Joo et al. (2016) fabricated random, semialigned, and highly aligned PCL fibres using two‐pole electrospinning to study attachment, proliferation, and migration of PC12 neuronal like cells. They observed both directional growth and elongation in PC12 neuronal cells when grown on PCL aligned fibres (Panahi‐Joo et al., 2016). In another study, neural stem cells displayed increased oligodendrocyte differentiation on 283‐nm fibres, whereas increased neuronal differentiation was observed when grown on 749‐nm fibres (Christopherson, Song, & Mao, 2009).

Although RN22 Schwann cells were scarcely detected, statistical analysis showed a significant increase in the number of NG108‐15 neuronal cells on the three substrates when grown in coculture with Schwann cells (Figure 6b). The number of neuronal cells increased significantly on medium fibres, large fibres, and PHA blend films when cocultured with Schwann cells. These findings suggest that Schwann cells are able to enhance neuronal cell growth significantly. This could be due to the secretion of a combination of neurotrophic factors (e.g., nerve growth factor and/or brain‐derived neurotrophic factor) from Schwann cells into the culture medium with paracrine signalling to the neuronal NG108‐15 cells.

## 5 CONCLUSIONS

Highly aligned and uniform fibres with varying diameters were successfully fabricated by controlling electrospinning parameters. Preliminary studies were carried out to evaluate the effect of fibres on the growth of NG108‐15 neuronal cells by live/dead tests. Cell migration observed on the electrospun fibres showed directional alignment in accordance with the direction of the fibres. The distribution of cells on the electrospun fibres had a more uniform aligned arrangement when compared with that on the surface of the PHA blend films. This finding agreed with previous studies in which electrospun fibres have been shown to affect cell proliferation, differentiation, and migration. Additionally, no cytotoxic effect was found when neuronal cells were grown on any of the studied substrates. Thereafter, the relationship between PHA blend microfibre diameter and neuronal growth under two conditions, individually and in coculture with RN22 Schwann cells, was evaluated. Results displayed from both single cell type and coculture studies revealed that all PHA blend fibre groups were not only able to support growth but also to guide aligned distribution of neuronal cells when grown individually and in the presence of RN22 Schwann cells. All the substrates, including the controls, were found to support neurite outgrowth. Results revealed a direct relationship between fibre diameter and neuronal growth and differentiation. The greatest number of neuronal cells was displayed on large fibres (13.50 ± 2.33 μm) when grown individually and in coculture. The number of NG108‐15 cells increased on all the substrates when cocultured with RN22 cells. Thus, aligned large fibre‐based constructs are a potential alternative for the efficient growth and differentiation of neuronal cells, especially in the context of inner structures of NGCs, which can act as highly efficient alternatives to the standard autografting procedures used to repair nerve gaps. In future, because the PHA blend fibres fabricated in this study have proven to be optimal guidance cues, these can be used as the lumen structures of NGCs. Varying microfibre scaffolds (2.0–13.0 μm) will be produced as internal structures within NGCs and implanted in 10‐mm defect gaps in median nerves, using rats as the animal model. Peripheral nerve regeneration will be assessed after 1, 3, and 6 months of implantation using morphological, morphoquantitative, and stereological analysis. Subject to positive results, clinical trials will be the final outcome.

## CONFLICT OF INTEREST

The authors declare no competing financial interest.

## Supporting information


**Figure S1.** Confocal micrographs of NG108‐15 neuronal cells ummunolabelled for beta‐III tubulin after four days in culture on aligned PHA blend fibres. (A, G), Neuronal cells inmunolabelled for beta‐III tubulin grown on small fibres. (B, H), Neuronal cells **inmunolabelled** for beta‐III tubulin grown on medium fibres. (C, I) Neuronal cells inmunolabelled for beta‐III tubulin on large fibres 2. (D, J), Neuronal cells ummunolabelled for beta‐III tubulin + DAPI grown on small fibres. (E, K), Neuronal cells inmunolabelled for beta‐III tubulin + DAPI grown on medium fibres. (F, L) Neuronal cells ummunolabelled for beta‐III tubulin + DAPI grown on large fibres. Aligned cellular growth was clearly observed on the three different fibre diameters. Scale bar = 12.5 μm.

**Figure S2.** Confocal micrographs of NG108‐15 neuronal cells ummunolabelled for beta‐III tubulin + DAPI after four days in culture on P(3HO)/P(3HB) blend flat film, PCL and glass.
A) Neuronal cells inmunolabelled for beta‐III tubulin grown on P(3HB)/P(3HO) blend flat film.
B) Neuronal cells inmunolabelled for beta‐III tubulin grown on PCL. C) Neuronal cells inmunolabelled for beta‐III tubulin grown on glass. D) Neuronal cells inmunolabelled for beta‐III tubulin + DAPI grown on P(3HB) blend flat film. E) Neuronal cells inmunolabelled for beta‐III tubulin + DAPI grown on PCL. F) Neuronal cells inmunolabelled for beta‐III tubulin + DAPI grown on glass. Cell growth was randomly oriented on each of the flat surfaces and clusters of neuronal cells connected through neurites were observed. Scale bar = 12.5 μm.
