# Supplementary figures and images for: Unidirectional neuronal cell growth and differentiation on aligned polyhydroxyalkanoate blend microfibres with varying diameters
Source: J Tissue Eng Regen Med. 2019 Jun 11;13(9):1581–94. doi: 10.1002/term.2911 (PMC6790610; doi:10.1002/term.2911)

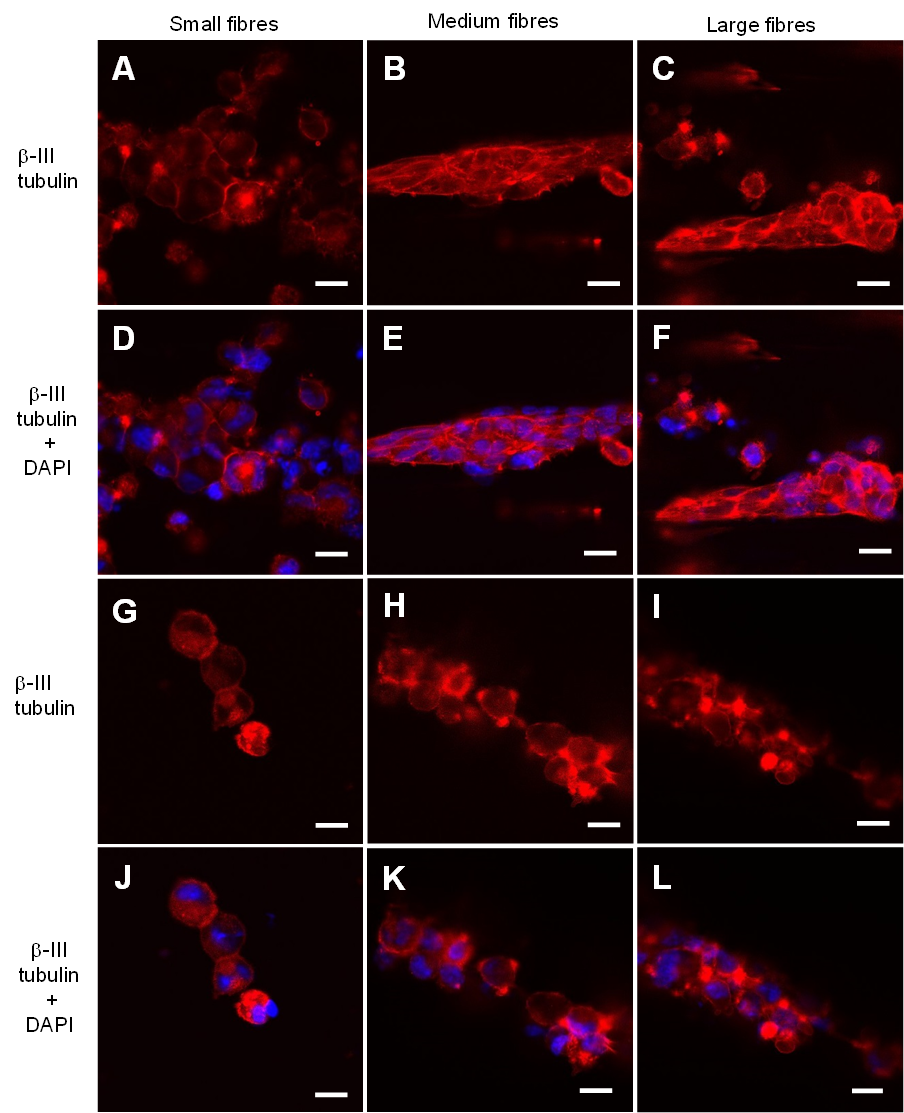

Supplement: Supplementary file 1 — Figure S1. Confocal micrographs of NG108‐15 neuronal cells ummunolabelled for beta‐III tubulin after four days in culture on aligned PHA blend fibres. (A, G), Neuronal cells inmunolabelled for beta‐III tubulin grown on small fibres. (B, H), Neuronal cells inmunolabelled for beta‐III tubulin grown on medium fibres. (C, I) Neuronal cells inmunolabelled for beta‐III tubulin on large fibres 2. (D, J), Neuronal cells ummunolabelled for beta‐III tubulin + DAPI grown on small fibres. (E, K), Neuronal cells inmunolabelled for beta‐III tubulin + DAPI grown on medium fibres. (F, L) Neuronal cells ummunolabelled for beta‐III tubulin + DAPI grown on large fibres. Aligned cellular growth was clearly observed on the three different fibre diameters. Scale bar = 12.5 μm. [file TERM-13-1581-s001.tif]

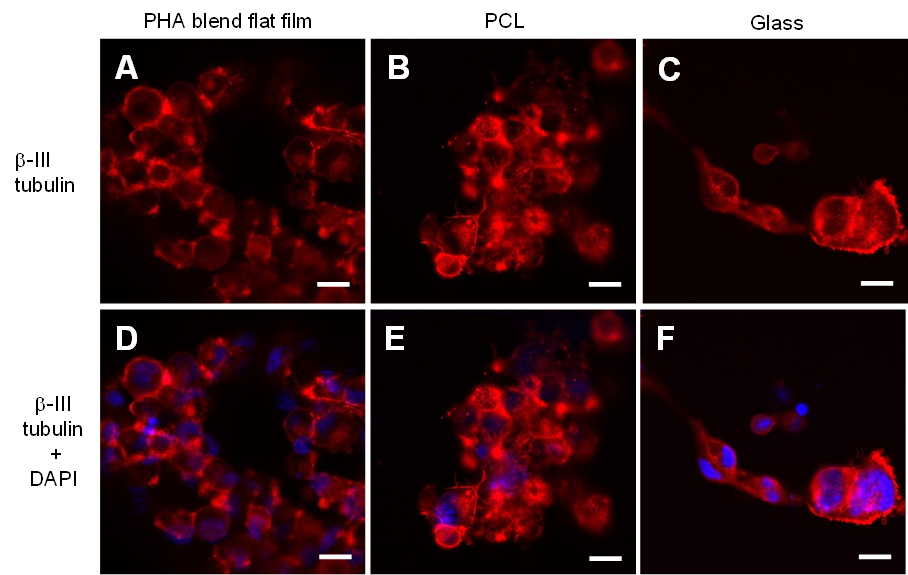

Supplement: Supplementary file 2 — Figure S2. Confocal micrographs of NG108‐15 neuronal cells ummunolabelled for beta‐III tubulin + DAPI after four days in culture on P(3HO)/P(3HB) blend flat film, PCL and glass. A) Neuronal cells inmunolabelled for beta‐III tubulin grown on P(3HB)/P(3HO) blend flat film. B) Neuronal cells inmunolabelled for beta‐III tubulin grown on PCL. C) Neuronal cells inmunolabelled for beta‐III tubulin grown on glass. D) Neuronal cells inmunolabelled for beta‐III tubulin + DAPI grown on P(3HB) blend flat film. E) Neuronal cells inmunolabelled for beta‐III tubulin + DAPI grown on PCL. F) Neuronal cells inmunolabelled for beta‐III tubulin + DAPI grown on glass. Cell growth was randomly oriented on each of the flat surfaces and clusters of neuronal cells connected through neurites were observed. Scale bar = 12.5 μm. [file TERM-13-1581-s002.tif]
